# Predictive value of immature granulocyte in the diagnosis of acute complicated appendicitis

**DOI:** 10.1371/journal.pone.0279316

**Published:** 2022-12-21

**Authors:** Gulsum Feyza Turkes, Abdulkadir Unsal, Hakan Bulus

**Affiliations:** 1 Department of Medical Biochemistry, Ankara Ataturk Sanatoryum Training and Research Hospital, Ankara, Turkey; 2 Department of General Surgery, Ankara Ataturk Sanatoryum Training and Research Hospital, Ankara, Turkey; Kyushu University Hospital, JAPAN

## Abstract

**Introduction:**

The goal in appendicitis is early diagnosis and early treatment. Complications develop as treatment is delayed. Therefore, there is a need for fast, low-cost markers that can be diagnosed earlier. The aim of this study is to show the effectiveness of immature granulocyte (IG) level in determining the complication of acute appendicitis.

**Method:**

In this retrospective cross-sectional study, 99 patients with acute appendicitis and 41 control groups were included in the study. Acute appendicitis patients were divided into two groups as acute simple appendicitis(n = 65) and acute complicated appendicitis(n = 34). In all groups, demographic data, immature granulocyte (IG) count(x10^3^/μL), IG ratio (IG%), white blood cell (WBC), polymorphonuclear leukocytes (PNL), lymphocyte, monocyte, platelet, mean platelet volume (MPV), ferritin levels were recorded. The blood analyses were performed the Mindray BC6800 automated hematology analyzer using standard laboratory protocols. All statistical testing was undertaken using IBM SPSS Statistics for Mac.

**Results:**

Compared to the acute simple appendicitis, acute complicated appendicitis patients had significantly higher levels WBC, PNL, monocyte, IG count, and IG% (p = 0.009, p = 0047, p = 0.001, p = 0.018; respectively) while there was no significant difference in ferritin between groups (p = 0.49). In the ROC analysis, AUC value was found for IG count and IG% (0.893 and 0.725, cut-off 0.005 and 0.05; respectively) for acute appendicitis. The negative predictive value of IG for Acute Appendicitis was 85% and was the same as that of WBC. In acute complicated appendicitis, the AUC for IG and IG% was 0.796 (cut-off 0.02) and 0.693 (cut-off 0.2), respectively. Positive Likelihood Ratio (+LR; 2.1) value of IG was found higher than other complete blood count (CBC) tests.

**Conclusion:**

The IG count is test with fast, more predictive value than another CBC tests, and without cost in the early diagnosis of acute complicated appendicitis. It is strong negative predictive test for Acute Appendicitis disease.

## Introduction

Acute appendicitis is a disease that requires surgery, mostly characterized by abdominal pain between the ages of 5 and 45. It has an approximately lifetime risk of 8.6% in men and 6.7% in women [1].

The diagnosis of acute appendicitis is made by computed tomography and doppler ultrasonography. The Alvarado scoring system is commonly used scoring systems to determine the need for surgical intervention. The simpler known scoring system is the Appendicitis Inflammatory Response Scoring system. The parameters in this scoring system are; vomiting, right lower quadrant pain, rebound and muscular defense, body temperature, polymorphonuclear leukocyte dominance (PNL), leukocyte count (WBC), c-reactive protein (CRP) level [2].

The goal in appendicitis is early diagnosis and early treatment. Uncomplicated appendicitis is simple appendicitis. Complications develop as treatment is delayed. Acute complicated appendicitis progresses with serious problems and causes postoperative complications, delayed recovery, increased hospital stay and increased cost [2]. As in previous studies, there is a need for faster, low-cost markers that can be diagnosed earlier [3–8].

Immature granulocytes are premature granulocytes (promyelocyte, myelocyte, metamyelocyte) released from the bone marrow when there is infection, inflammation, sepsis, and other stimuli. As a result of the bone marrow produces more leukocytes and immature granulocytes migrate to the peripheral blood, a left shift is observed in the origin of leukocytes [9]. Band forms of neutrophils are more mature neutrophil precursors than promyelocyte, myelocyte, and metamyelocyte. In order to show infection earlier, band forms are examined in peripheral smear, but among evaluators variability is high. The standard deviation and repeatability are poor [10, 11]. Therefore, immature granulocyte counting has been developed in automated blood counting devices, which is fast, does not require extra samples, and has good reproducibility [9].

In our study, it was aimed to evaluate effectiveness of immature granulocyte (IG) count and IG ratio (IG%), in distinguishing complicated and simple acute appendicitis.

## Materials and methods

This research was designed as a retrospective study and was conducted between January 2021 and October 2021. The study initially included 150 individuals between 18 and 65 years of age who presented for diagnosis the surgeon clinics in Ankara Ataturk Sanatoryum Training and Research Hospital Biochemistry Laboratory in Ankara in Turkey. This study was conducted with the approval of the “Ankara Kecioren Training and Research Hospital Clinical Research Ethics Committee” (2012-KAEK-15/2421). Informed voluntary consent form was obtained from the patients as a routine procedure before the surgery to use the data in scientific studies. OpenEpi software was used for sample size analysis based on a 95% confidence interval and 80% power.

Patients with pregnancy, receiving blood transfusions, using immunsupresif or steroid, hematologic malignancy and patients with missing data were excluded from the study. After excluding patients with neoplasm, the study was conducted with a total of 140 individual consisting of 41 healthy individual (control group) and 99 appendicitis patients. Based on surgical findings and histopathology reports, the patients were classified into 3 groups as control group, simple appendicitis, and complicated appendicitis (perforated, abscess, and peritonitis).

Complete blood count (CBC), ferritin levels were analysed by routine laboratory methods. IG count was measured by DIFF scattergram method from whole blood samples (Mindray BC-6800, China. IG ratio is calculated IG count. The IG ratio is also called the Delta-Neutrophil Index (DNI). Neutrophil to lymphocyte ratio (NLR), lymphocyte to monocyte ratio (LMR) were calculated these parameters.

### Statistical analysis

All data were analysed by IBM SPSS Statistics for Mac, version 26.0 for Mac OS X (IBM Corp., Armonk, N.Y., USA). Parameters were evaluated for normal distribution with the Kolmogorov–Smirnov test. Correlation analyses were done using Spearman’s test. The categorical values of the patients were expressed as a number and a percentage and were analysed with a Chi-square test. Continued values were presented as a median value and an interquartile range (IQR) of 25%–75%. In univariate analysis, Mann-Whitney U test was used to compare the means of continuous data. For post-hoc analysis, new p-value level calculated with Bonferroni correction. The 95% confidence intervals (95% CIs) were also calculated when appropriate, and a p-value <0.05 was considered statistically significant.

## Results

The study group included a total of 140 adults between the ages of 18 and 65 years. In terms of sex distribution, 55 (55.6%) of the Acute Appendicitis group were men, compared to 15 men (36.6%) in the Control group. Demographic and laboratory data for the acute appendicitis and control groups are shown in Table 1. Compared to the control group, Acute appendicitis patients had significantly higher levels WBC, neutrophil, monocyte, MPV, IG, and IG%.

**Table 1:** Biochemical variables of control and acute appendicitis groups.

	Control Group (n = 41)		Acute Appendicitis (n = 99)		
	Median	IQR (25–75)	Median	IQR (25–75)	P value
Age	43	33–57	33	26–45	0.003**
WBC (x10^3^/μL)	6.8	6.3–7.7	14.5	12–16.8	<0.001*
Neutrophil (x10^3^/μL)	3.98	3.5–4.7	11.5	8.7–14	<0.001*
Lymphocyte (x10^3^/μL)	2.46	2–2.7	2.1	1.4–2.8	0.088
Monocyte (x10^3^/μL)	0.42	0.32–0.48	0.69	0.52–0.95	<0.001*
Platelet (x10^3^/μL)	258	214–305	248	215–283	0.349
MPV (fL)	9.6	8.9–10.3	10.3	9.5–10.9	0.003**
NLR	1.8	1.5–2.4	5.6	3–8.5	<0.001*
LMR	5.6	4.8–6.9	2.9	2.1–2.9	<0.001*
Neutrophil to IG ratio	330	0–445	473	359–678	<0.001*
IG (x10^3^/μL)	0.01	0–0.01	0.02	0.01–0.03	<0.001*
IG (%)	0.1	0–0.1	0.1	0.1–0.2	<0.001*
Ferritin (μg/L)	33.9	20.53–65.21	71.3	29.3–143.1	0.07

* p <0.001

** p <0.05

Sex-based comparison of IG levels in the entire study group showed that IG levels were significantly higher in men than in women [0.02 (0.01–0.03) vs 0.01 (0.01–0.02)]; respectively, p = 0.007).

Demographic and laboratory data for the acute simple appendicitis and acute complicated appendicitis are shown in Table 2. Compared to the acute simple appendicitis, Acute complicated appendicitis patients had significantly higher levels WBC, PNL, monocyte, IG, and IG%.

**Table 2:** Biochemical variables of control and acute appendicitis groups.

	Acute Simple Appendicitis (n = 65)		Acute Complicated Appendicitis (n = 34)		
	Median	IQR (25–75)	Median	IQR (25–75)	P value
Age	33	24–42	36	29–49	0.09
WBC (x10^3^/μL)	13.6	11.7–16.1	15.75	13.5–18	0.009*
Neutrophil (x10^3^/μL)	10.7	8.4–13	12.2	9.7–14.7	0.047*
Lymphocyte (x10^3^/μL)	2	1.4–2.7	2.2	1.6–2.9	0.401
Monocyte (x10^3^/μL)	0.61	0.49–0.8	0.81	0.68–1.01	0.001*
NLR	5	2.6–9.2	6	4.5–7.4	0.336
LMR	3	2.4–4.4	2.4	1.9–3.4	0.024*
Neutrophil to IG ratio	510	370–650	442	284–750	0.704
Platelet (x10^3^/μL)	247	215–283	252	219–283	0.985
MPV (fL)	10.3	9.4–10.8	10.2	9.5–11	0.693
IG (x10^3^/μL)	0.02	0.01–0.03	0.03	0.02–0.05	0.001*
IG (%)	0.1	0.1–0.2	0.2	0.1–0.3	0.018*
Ferritin (μg/L)	84.2	38–144.6	29.6	27.1–137	0.49

* p <0.05

The data obtained in separate correlation analysis of the control and acute appendicitis groups are presented in Table 3.

**Table 3:** Correlation analysis in control and acute appendicitis groups.

Control Group		WBC	Neutrophil	Lymphocyte	Monocyte	MPV
IG (x10^3^/μL)	r	-0.059	0.033	-0.056	-0.010	-0.245
	p	0.716	0.835	0.726	0.948	0.123
IG (%)	r	-0.109	-0.136	-0.078	-0.036	-0.327*
	p	0.498	0.395	0.629	0.823	0.037
**Acute Appendicitis**		**WBC**	**Neutrophil**	**Lymphocyte**	**Monocyte**	**MPV**
IG (x10^3^/μL)	r	0.522*	0.515*	-0.007	0.414*	0.119
	p	<0.001	<0.001	0.943	<0.001	0.239
IG (%)	r	0.160	0.193	-0.194	0.190	0.079
	p	0.113	0.056	0.054	0.060	0.439

* Middle degree correlation

**Fig 1:**
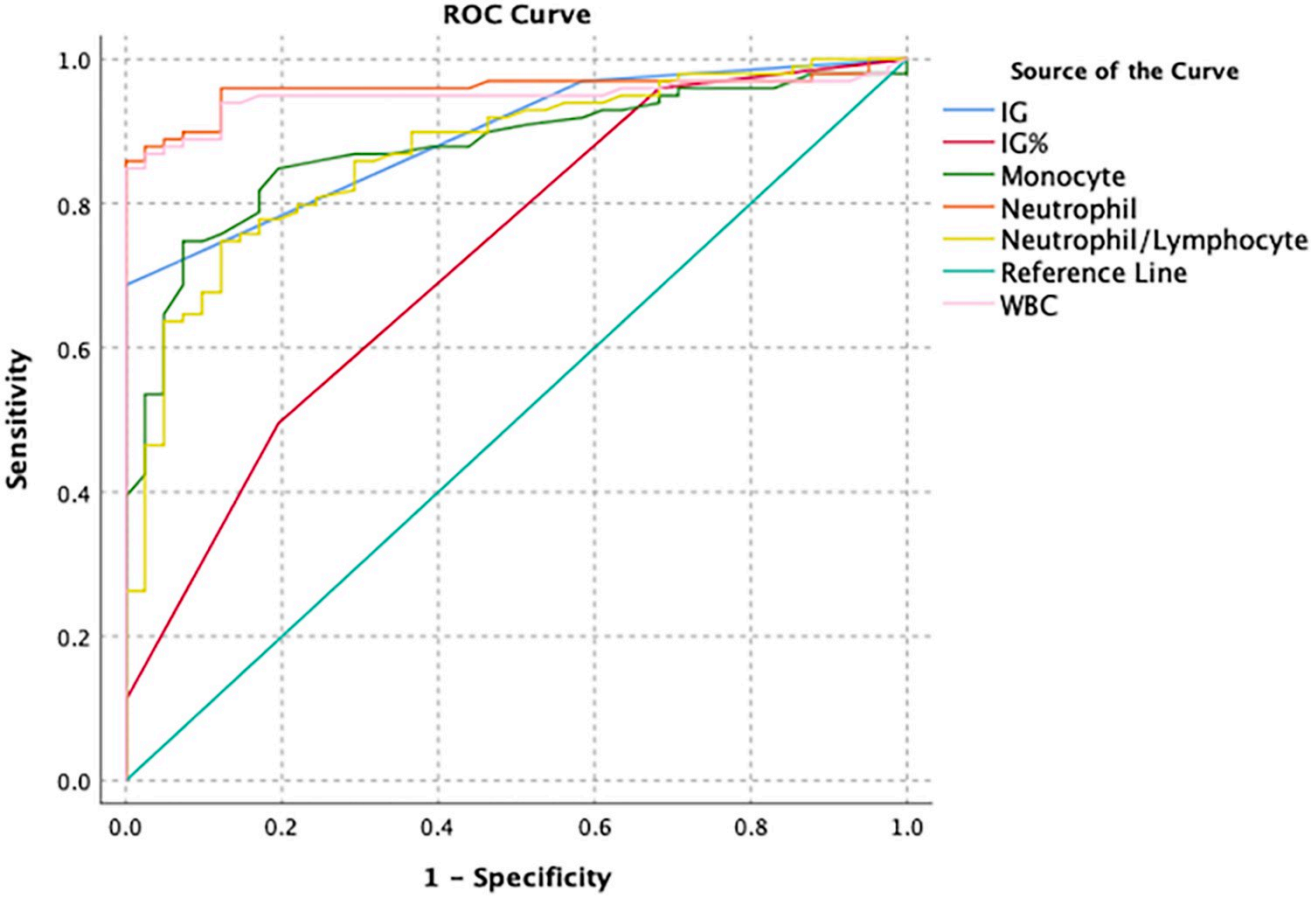
ROC analysis of haematological parameters in the diagnosis of acute appendicitis patients.

**Fig 2:**
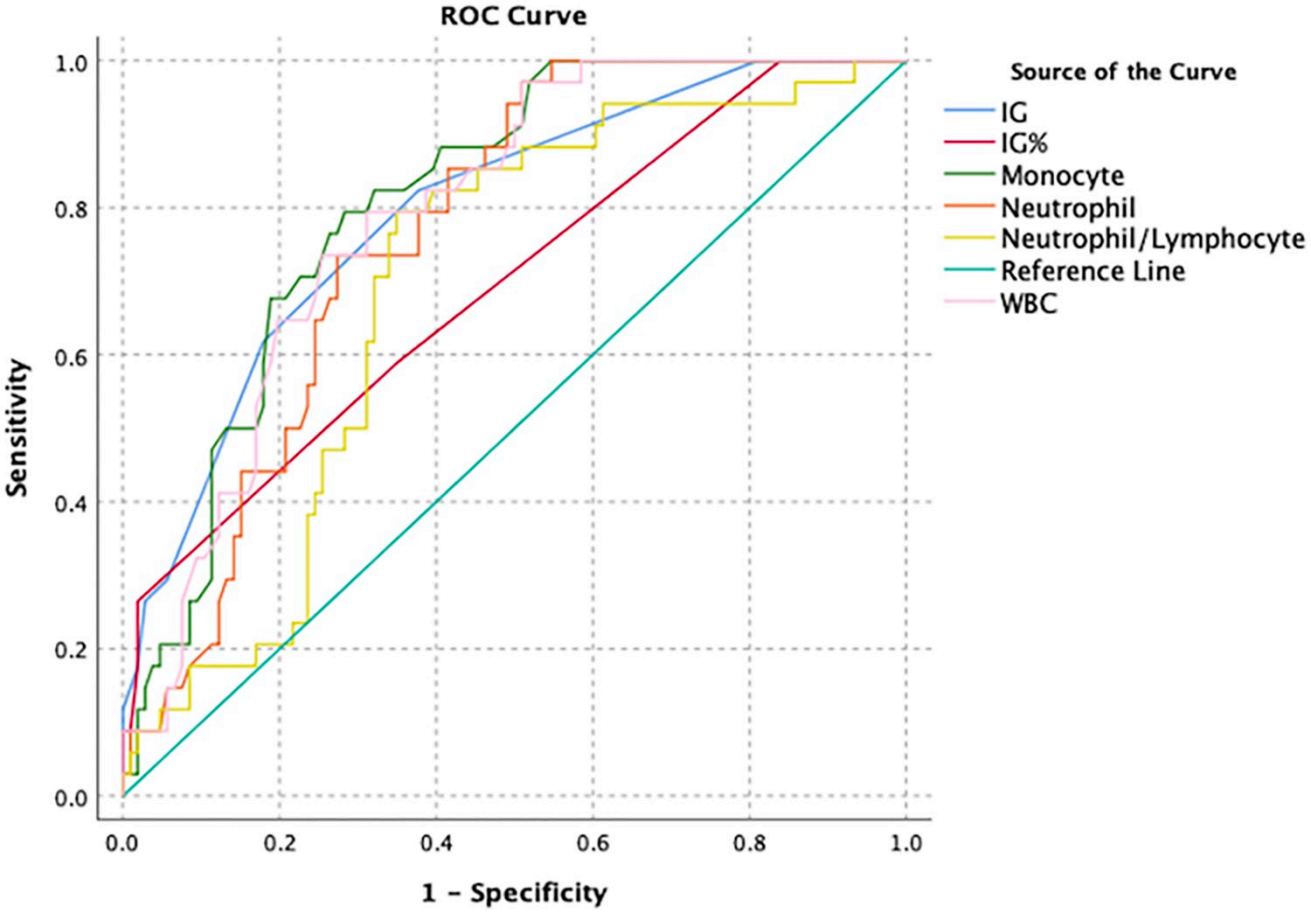
ROC analysis of haematological parameters in the diagnosis of acute complicated appendicitis patients.

In the ROC analysis, AUC value was found for IG and IG% (0.893 and 0.725, cut-off 0.005 and 0.05; respectively) for acute appendicitis (Fig 1). In acute complicated appendicitis, the AUC for IG and IG% was 0.796 (cut-off 0.02) and 0.693 (cut-off 0.2), respectively (Fig 2).

The predictive parameters related to diagnostic of acute appendicitis disease are in the Table 4. The sensitivity and specificity values were 97% and 41.5% for IG, with a cut-off value of 0.005. The sensitivity and specificity values were 89.9% and 90.2% for neutrophil, with a cut-off value of 6.225. The IG is the best negative likelihood ratio (-LR) (0.08) for disease and it has moderate effect rating (only -LR < 0.1). The IG count is for ruling-out, but not ruling in. Although The neutrophil is statistically significant, it isn’t high clinical validity [12].

**Table 4:** Predictive evaluation of the best parameters to show diagnostic of disease.

	Cut-off	Sensitivity (%)	Specificity (%)	AUC	%95 CI	+LR	-LR	PPV (%)	NPV (%)	p
IG	0.005	97	41.5	0.893	0.843–0.944	1.66	0.08	80	85	<0.001
IG%	0.05	96	31.7	0.725	0.632–0.817	1.4	0.13	77	76	<0.001
NLR	2.62	78.8	78	0.869	0.808–0.931	3.6	0.27	87	65	<0.001
WBC	9.6	88.9	87.8	0.948	0.909–0.986	7.3	0.13	94	85	<0.001
Neutrophil	6.225	89.9	90.2	0.958	0.924–0.992	9.2	0.11	95	80	<0.001
Monocyte	0.495	81.8	82.9	0.876	0.818–0.934	4.8	0.22	82	65	<0.001

The predictive parameters related to complicated of acute appendicitis disease are in the Table 5. The sensitivity and specificity values were 61.8% and 70.8% for IG, with a cut-off value of 0.03. The IG is the best positive LR (+LR) and -LR, but it isn’t high clinical validity. The IG test not good for ruling-in and ruling-out [12].

**Table 5:** Predictive evaluation of the best parameters to show complication of disease.

	Cut-off	Sensitivity (%)	Specificity (%)	AUC	%95 CI	+LR	-LR	PPV (%)	NPV (%)	p
IG	0.03	61.8	70.8	0.700	0.589–0.810	2.1	0.5	62.5	71	0.001
IG%	0.2	58.8	55.4	0.633	0.514–0.752	1.3	0.7	75	79	0.030
NLR	5.545	58.8	52.3	0.559	0.443–0.675	1.2	0.8	77	67	0.336
WBC	14.85	64.7	63.7	0.661	0.549–0.773	1.8	0.6	47	78	0.009
Neutrophil	11.65	64.7	60	0.622	0.509–0.735	1.6	0.6	40	78	0.047
Monocyte	0.725	67.6	67.7	0.708	0.605–0.811	1.1	0.5	47	79	0.001

## Discussion

Acute appendicitis is the most common surgical cause of acute abdomen. The goal in acute appendicitis is early diagnosis and early treatment. Complications develop as treatment is delayed. Acute complicated appendicitis progresses with serious problems and causes postoperative complications, delayed recovery, increased hospital stay and increased cost [2]. CT scan, MRI, and USG are commonly used in the diagnosis of acute appendicitis. However, these methods are costly. In addition, CT scan should not be used in children, pregnant women and patients with GFR ≤ 30 mL/min [13]. The IG is simple, inexpensive, and readily available parameters which can be obtained from CBC in all patients with acute appendicitis. In our study it was concluded that elevated IG count were associated with risk for complication in acute appendicitis. We think that they can be used to predict complications in patients with definitive diagnosis of acute appendicitis.

Studies have shown that acute appendicitis is more risky in men [2]. In our study, 55.6% of patients with acute appendicitis were male. In our study, WBC, neutrophil, monocyte, MPV, NLR levels were found to be higher in patients with acute appendicitis than control group, while LMR levels were found to be lower in patients with acute appendicitis. Our findings were consistent with the literature [3, 4, 7].

Immature granulocytes are premature granulocytes (promyelocyte, myelocyte, metamyelocyte) released from the bone marrow when there is infection, inflammation, sepsis and other stimuli. As a result of the bone marrow produces more leukocytes and immature granulocytes migrate to the peripheral blood, a left shift is observed in the origin of leukocytes [9]. In previous studies, immature granulocytes were found to be significantly higher in inflammatory conditions such as pancreatitis, sickle cell disease, liver abscess, and infective complications after cardiac surgery [14–17]. Kilercik et al. also found significantly higher IG levels in Covid 19 patients [18]. In different studies are IG and IG% are statistically significantly higher acute appendicitis than in the control group and acute complicated appendicitis group than in the acute simple appendicitis group [3, 4, 7]. In our study, IG count and IG% were significantly higher in acute complicated appendicitis than in acute simple appendicitis.

Nonoperative approaches are available for the acute simple appendicitis are available. Nonoperative treatment is a strategy in which patients receive antibiotics with the aim of avoiding surgery but it may be difficult to diagnose simple appendicitis because there are not enough markers [19]. A significant increase in the IG count and the IG% in acute complicated appendicitis may play an important role in the differential diagnosis of acute simple appendicitis and acute complicated appendicitis. In this case, non-operative current approaches can be chosen more easily in the treatment.

In the pathophysiology of acute appendicitis, it is presumed that there is obstruction in the appendix orifice. Decreased blood flow in the hypoxic-ischemic area leads to anaerobic metabolism with the formation of free oxygen radicals by the reduction of free metals and the catalyst effect of the superoxide dismutase enzyme. This results in increased IMA levels in blood [20]. Bacterial proliferation occurs in the obstructed appendix as the disease progresses [1]. Senthilnayagam et al. found the optimal cut-off in IG and IG% in 200 patients with bacterial infection as 0.03x103/μL and 0.5%, respectively [21]. In our study, we found the optimal cut-off in IG and IG% in patients with acute complicated appendicitis as 0.03x10^3^/μL and 0.2%, respectively.

It is understood that there is no specific test as it is known that immature granulocyte increases in different inflammatory conditions. The sensitivity and specificity of the Alvarado scoring system used in acute appendicitis diagnosis were 93.5% and 80.6%, respectively [22]. The sensitivity and specificity of the Appendicitis Inflammatory Response System was determined as 90% and 55%, respectively [23]. In our study, although the sensitivity of IG and IG% was 97% and 96%, respectively, for acute appendicitis patients, the specificity was 41.5% and 31.7%, respectively. In a meta-analysis, the rates of detection of normal appendixes on CT scan, MRI, and US was 84%, 69%, and 71%, respectively [24]. In our study, NPV (85%) of IG was found at the same rate as WBC in patients with appendicitis. -LR was determined as the strongest according to other hematological parameters. Moreover, the negative predictive value (NPV) of IG in our study was found to be close to the NPV of CT scan in the meta-analysis. In a meta-analysis conducted by Park et al., DNI (IG%) was found to be a predictive and prognostic test in patients with sepsis [25]. Guler et al., it was found that the positive predictive value of the IG count was higher for patients with a positive diagnosis of acute appendicitis according to the CT scan result when compared to other hematological parameters [26]. In our study, we classified appendicitis patients according to the surgical findings and histopathology reports, not according to the tomography findings. We observed that the positive predictive value (PPV) and +LR of the IG count were higher than the other hematological parameters in patients with acute complicated appendicitis. According to our study, we can say that IG count is more powerful than other CBC tests in excluding appendicitis and detecting appendicitis complications.

This study has limitations. The major limitation is the retrospective design of the study. In addition, it was conducted in a single center with relatively small sample size. Moreover, clinical symptoms, physical examination and other laboratory data were not analysed in this study.

## Conclusions

Our study shows that the IG count is strong negative predictive test for acute appendicitis disease, and it is more predictive value than another CBC test for acute complicated appendicitis. If the IG count is differentiated from simple appendicitis and complicated appendicitis, non-operative treatment approaches can be performed. The IG count can be a strong and inexpensive test method however large-scale prospective studies are needed.

## Supporting information

S1 Dataset (XLSX)
